# Extracellular Vesicle Encapsulated MicroRNAs in Patients with Type 2 Diabetes Are Affected by Metformin Treatment

**DOI:** 10.3390/jcm8050617

**Published:** 2019-05-07

**Authors:** Vikas Ghai, Taek-Kyun Kim, Alton Etheridge, Trine Nielsen, Torben Hansen, Oluf Pedersen, David Galas, Kai Wang

**Affiliations:** 1 Institute for Systems Biology, Seattle, WA 98109, USA; vghai@systemsbiology.org (V.G.); tkim@systemsbiology.org (T.-K.K.); 2 Pacific Northwest Research Institute, Seattle, WA 98103, USA; aetheridge@pnri.org (A.E.); djgalas@gmail.com (D.G.); 3 Novo Nordisk Foundation Center for Basic Metabolic Research, Faculty of Health and Medical Science, University of Copenhagen, 1017 Copenhagen, Denmark; trine.nielsen@sund.ku.dk (T.N.); torben.hansen@sund.ku.dk (T.H.); oluf@sund.ku.dk (O.P.)

**Keywords:** microRNAs, extracellular vesicles, type 2 diabetes, metformin

## Abstract

Recently, microRNAs (miRNAs) in circulating extracellular vesicles (EVs), have emerged as a source of potential biomarkers for various pathophysiological conditions, including metabolic disorders such as diabetes. Type 2 diabetes mellitus (T2DM), is the most prevalent form of diabetes in the USA, with 30 million diagnosed patients. Identifying miRNA biomarkers that can be used to assess response to glucose lowering treatments would be useful. Using patient plasma samples from a subset of the Danish Metagenomics of the Human Intestinal Tract (MetaHIT) cohort, we characterized miRNAs from whole plasma, plasma-derived EVs, and EV-depleted plasma by small RNA-sequencing to identify T2DM associated miRNAs. We identified several miRNAs that exhibited concentration changes between controls and non-metformin treated T2DM patients and we validated a subset of these by quantitative reverse transcription-polymerase chain reaction (qRT-PCR). The results showed that the concentrations of many T2DM-affected miRNAs in EV (but not in whole or EV-depleted plasma) decreased to levels close to those of healthy controls following metformin treatment. Among other potential uses of these differentially expressed miRNAs, some might be useful in assessing the response to metformin in T2DM patients.

## 1. Introduction

Diabetes is a major health epidemic accounting for a significant proportion of health-care-related expenditures in many countries [1]. In the United States alone, there are about 30 million patients living with diabetes, the majority with type 2 diabetes mellitus (T2DM) [2]. Elevated blood glucose is the hallmark of T2DM, which eventually leads to micro- and macro-vascular complications of major organs, among other effects. Onset of T2DM is usually preceded by a prediabetic phase, where blood glucose levels are elevated but below the level for T2DM. In many cases prediabetes results from development of insulin resistance (IR) of glucose metabolizing tissues which will eventually lead to pancreatic β-cell dysfunction. Managing the blood glucose level through exercise, dietary change, and drug treatment are the principal approaches to T2DM management. A drug to treat T2DM is metformin, which primarily reduces hepatic gluconeogenesis thereby lowering tissue and circulating levels of glucose [3].

In recent years, cell-free microRNAs (miRNAs) present in various body fluids, including those packaged in extracellular vesicles (EVs), have emerged as potential biomarkers for various diseases and health conditions, including T2DM [3]. There have been several studies examining the changes of circulating miRNAs associated with T2DM. One of the earliest studies identified several T2DM affected circulating miRNAs, and found the plasma miR-126-5p level is changed in both T2DM patients and mouse models [4]. Other studies have also observed the association of miR-126-5p level in plasma with insulin treatment [5,6,7]. Additional T2DM associated circulating miRNAs, such as miR-30d-5p [6,7,8], miR-122-5p [9,10,11], miR-146a-5p [8,12], miR-192-5p [13,14], and miR-375-5p [7,8] have also been reported. Several of these miRNAs, such as miR-30d-5p [9], miR-122-5p [9], miR-126-5p [5], and miR-192-5p [15] have also been associated with prediabetes. While these circulating miRNAs show promise for identifying individuals with T2DM, the results of our meta-analysis showed many inconsistent findings among studies, which may have been caused by differences in sample preparation methods or miRNA measurement platforms [16]. All miRNA measurement platforms have advantages and disadvantages. For example, next generation sequencing (NGS) based small-RNA sequencing (sRNAseq) provides higher dynamic range and allows for the analysis of all potential miRNAs, and even identification of novel miRNAs, when compared to quantitative reverse transcription-polymerase chain reaction (qRT-PCR), or microarray making it a better method for discovery-based studies. On the other hand, sRNAseq protocols, particularly the commercial library construction kits, are prone to severe sequence-specific biases. To address this, a modified small RNA sequencing library construction method, as well as a comprehensive data analysis pipeline, that allows for significant improvement of miRNA analysis from biological samples were developed [17,18]. This improved approach has been used in several diabetes related studies, including the identification of circulating miRNAs associated with the progression of T2DM from prediabetic individuals [19,20].

Many circulating miRNA studies do not consider EVs in circulation as a separate sample type rather using whole biofluids, such as plasma and serum. Those few EV based studies often use isolation methods such as ultracentrifugation or precipitation, which may alter their properties and co-purify RNAs associated with RNA-binding proteins or lipoproteins [21,22]. There are some technical advancements to improve the reproducibility of circulating miRNA studies in recent years. These including a size exclusion chromatography (SEC) based EV purification, an improved small RNA library construction method [18], and better tools to align short RNA sequence reads [17]. In the current study, we analyzed the small RNA spectra from whole plasma, plasma EVs and EV-depleted plasma obtained from T2DM patients (with or without metformin treatment) and from healthy control individuals who participated in the Danish part of the Metagenomics of the Human Intestinal Tract (MetaHIT) study [23].

Several circulating miRNAs including some EV-encapsulated miRNAs that are associated with T2DM or affected by metformin treatment were identified. These miRNAs may serve as possible biomarkers, assessing metformin treatment response in patients with T2DM.

## 2. Materials and Methods

### 2.1. Sample and Study Collection

Fasting plasma samples were obtained from a subset of the Danish MetaHIT cohort [23]. In the present study we included 80 individuals: 39 controls, 10 T2DM (non-metformin treated), and 31 T2DM (treated with metformin). Samples were matched for age, and body mass index (BMI) (Supplemental Table S1). Anthropometrics and biochemical measurements were obtained as previously described [24]. Plasma glucose was analyzed by a glucose oxidase method (Granutest, Merck, Darmstadt, Germany). HbA1c was measured on TOSOH G7 by ion-exchange high performance liquid chromatography. Serum insulin (excluding des-31,32-proinsulin and intact proinsulin) was measured using the AutoDELFIA insulin kit (Perkin-Elmer, Wallac, Turku, Finland). HOMA-IR was calculated as: (fasting plasma glucose (mmol/L) × fasting serum insulin (mU/L))/22.514. BMI was calculated as weight (kg) divided by height (m^2^) and obesity defined as BMI > 30 kg/m^2^. Plasma samples were collected after an overnight fast into EDTA-treated tubes and spun at 1000× *g* at 4 °C for 10 min to remove blood cells, and then 2000× *g* at 4 °C for 15 min to remove platelets and stored at −80 °C. Before RNA isolation, the plasma samples were spun at 10,000× *g* at 4 °C for 10 min to remove any remaining cellular debris and platelets. Prior to EV and RNA isolation, the plasma samples were spun at 10,000× *g* at 4 °C for 10 min to remove platelets and cellular debris.

### 2.2. Isolation of EV and EV-Depleted Fractions

EVs were isolated from 200 µL of plasma using size-exclusion chromatography (SEC) (qEV column, Izon Science, Cambridge, MA, USA) with de-gassed PBS as previously described [25]. Briefly, eluate fractions (∼500 µL each fraction) containing EVs (fractions 7–10) were collected and the subsequent fractions (11–35) were also collected as EV-depleted fractions. To confirm the purification of EVs from samples, the SEC-purified EVs were quantified using the qNano particle counter (Izon Science, Cambridge, MA, USA) using a NP150 pore, and visualized with transmission electron microscopy as previously described [21].

### 2.3. RNA Isolation and Small RNA Sequencing Library Construction

Circulating RNA was isolated from whole plasma, EVs, and EV-depleted fractions using the miRNeasy kit according to the manufacturer’s instructions. The RNA was eluted in 14 µL nuclease-free H_2_O, and the quantity and quality were assessed using a Bioanalyzer pico chip (Agilent Technologies, Santa Clara, CA, USA). To analyze circulating miRNA, we used a modified small-RNA library construction protocol to reduce small RNA library construction associated bias [18]. Individual library concentrations were assessed by NEBNext Library Quant Kit for Illumina (New England Biolabs, Ipswich MA), pooled (2 nM final concentration) and then run on a NextSeq500 sequencer (Illumina, San Diego, CA, USA).

### 2.4. Data Analysis

Sequence files (FASTQ format) were processed with an in-house small RNA analysis pipeline—sRNAnalyzer [17]. Briefly, the adapters were trimmed from the sequence reads, low complexity, low quality, and short reads (less than 15 nucleotides) were removed. The processed reads (reads that passed filtering) were then mapped against miRBase (www.mirbase.org) v21. The mapping was performed with single assignment and zero mis-matches allowed. The miRNA mapping results were normalized using read count per million of processed read and then log2 transformed. Based on the results, several invariant miRNAs (miRNAs that had a low sample to sample coefficient of variance across all samples, including plasma, EVs, and EV-depleted plasma) were identified, including miR-451a-5p and miR-486-5p. To be considered for analysis, a given miRNA had to have greater than 10 reads in at least 50% of samples, across all samples, including plasma, EVs, and EV-depleted plasma. To be considered to have a significant concentration change, a miRNA required a fold change greater than 1.5 (or a log2 fold change > ±0.60) with a *p*-value < 0.05 (calculated using the Wilcoxon rank sum test and corrected for multiple hypothesis testing using Benjamini-Hochberg). The miRTar database (*mirtar.mbc.nctu.edu.tw/*) was used to identify miRNA-mRNA interaction targets to determine biological processes that may be regulated by specific miRNA. In this approach, we required that each miRNA target must be validated by at least two different experimental methods. Gene enrichment analysis was performed with DAVID (Database for Annotation, Visualization and Integrated Discovery, https://david.ncifcrf.gov/), using default settings. Enrichment was based on the Benjamini-Hochberg corrected *p*-values for KEGG terms.

### 2.5. qRT-PCR

Quantitative Reverse Transcription Polymerase Chain Reaction (qRT-PCR) validation of miRNAs was performed using TaqMan Advanced miRNA assays (Thermo Fisher, Waltham, MA, USA). MiR-451a-5p and miR-486-5p were used as normalizers, since they were identified as invariant miRNAs in the sample set (low coefficient of variance across samples) based on the miRNA mapping results. Relative miRNA values are presented by ΔΔC_t_ values (Ct _reference_ − Ct _target_) [26].

### 2.6. Ethics

The study was approved by the Ethical Committees of the Capital Region of Denmark (HC-2008-017 and H-15000306) and was in accordance with the principles of the Declaration of Helsinki. All individuals gave written informed consent before participating in the study.

## 3. Results

### 3.1. Isolation of EVs from Fasting Plasma Samples of Healthy Controls and T2DM

**Figure 1:**
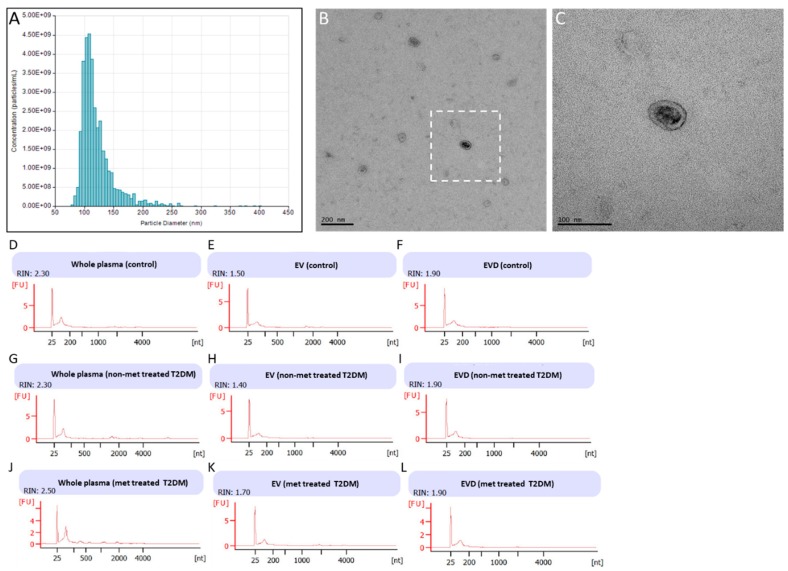
Isolation and characterization of extracellular vesicles (EVs) and RNA from plasma. (**A**) Histogram of EV isolated from plasma (**B**,**C**) EV imaged using TEM (**D**–**J**) bioanalyzer traces from whole plasma, EV, and EV-depleted (EVD) samples. (**A**) Histogram generated by qNano particle tracker of EVs isolated using SEC from patient plasma samples, with an average particle size of 122 nm. (**B**) Electron micrographs taken from isolated EVs, showing particles of a similar size that have characteristic shape and form of EVs. Black bar represents 200 nm scale. (**C**) Enlargement of boxed in area in (**B**). Black bar represents 100 nm scale. (**D**–**L**) Agilent RNA bioanalyzer traces of RNA isolated from control plasma (**D**), control EVs (**E**), control EVD (**F**), non-metformin (non-met) treated T2DM plasma (**G**), non-metformin treated type 2 diabetes mellitus (T2DM) EVs (**H**), non-metformin treated T2DM EVD (**I**), metformin (met) treated T2DM plasma (**J**), metformin treated T2DM EVs (**K**), and metformin treated T2DM EVD (**L**).

From the 80 individuals in the discovery set, 39 were healthy controls, 10 were non-metformin treated T2DM, and 31 were metformin-treated T2DM patients (Supplemental Table S1). The samples were matched with respect to gender, age and BMI, and showed no significant differences between the groups (Supplemental Table S1). To explore the possible changes in circulating miRNA profiles among the groups, we used next generation sequencing (NGS) platform to characterize the miRNA spectra in whole plasma, EVs, and EV-depleted plasma for each patient. EVs isolated from plasma had an average size of 122 nm, with an estimated average concentration of 4.24 × 10^8^ EVs/mL (Figure 1A). Electron microscopy images of EVs from these samples showed the presence of vesicles of ~100 nm in diameter with typical morphology (Figure 1B,C). The EVs, along with whole plasma and EV-depleted plasma, were then used to isolate RNA for small RNA analysis (Figure 1D–L).

### 3.2. MiRNA Levels from Small RNA Sequencing

About 10 million reads on average across all samples were obtained, among them approximately 7 million reads passed the pre-processing step (removal of adapter sequences and short or low complexity reads) (Table 1). In plasma, about 1.1 million processed reads mapped to various human miRNAs, while on average 293,000 and 634,000 reads mapped to various miRNAs in EV and EV-depleted samples, respectively (Table 1). In whole plasma, EVs, and EV-depleted plasma on average 693, 423, and 617 different miRNAs were observed (with at least one mapped read) in each sample, respectively. Among these observed miRNAs in whole plasma, EVs, and EV-depleted plasma, 446, 242, and 352 miRNAs had 10 or more mapped reads, respectively (Table 1).

### 3.3. miRNAs Associated with T2DM

**Figure 2:**
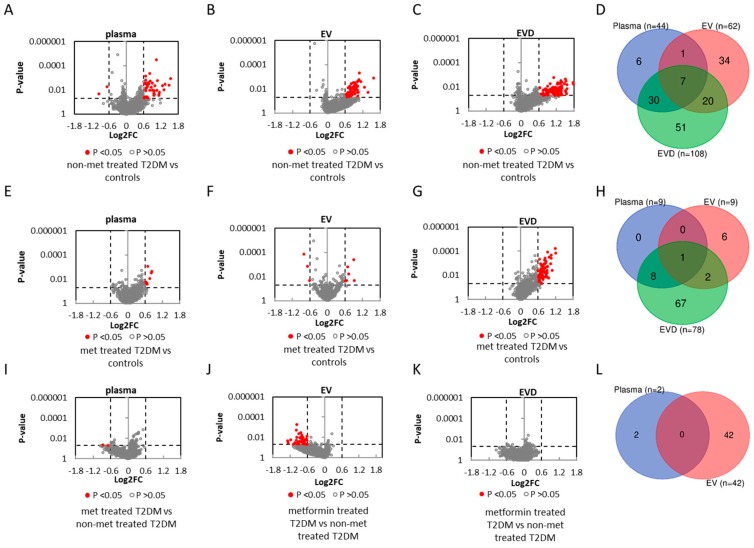
miRNAs with concentration changes between T2DM patients and healthy controls. (**A**–**C**) volcano plots for non-metformin (non-met) treated T2DM versus controls. (**E**–**G**) volcano plots for metformin (met) treated T2DM versus controls. (**I**–**K**) volcano plots for metformin treated versus non-metformin treated T2DM. Volcano plots showing miRNA log2 fold change (log2FC) versus *p*-value between non-metformin treated T2DM and control samples in plasma (**A**), EVs (**B**), EV-depleted (EVD) fractions (**C**), and (**D**) overlap between miRNAs that show concentration differences in plasma, EVs, and EVD fractions. Volcano plots showing miRNA log2 fold change (log2FC) versus *p*-value between treated T2DM and control in plasma (**E**), EVs (**F**), EVD fractions (**G**), and (**H**) overlap between miRNAs that show concentration differences in plasma, EVs, and EVD fractions. Volcano plots showing miRNA log2 fold change (log2FC) versus *p*-value between metformin treated T2DM and non-metformin treated T2DM in plasma (**I**), EVs (**J**), EVD fractions (**K**), and (**L**) overlap between miRNAs that show concentration differences in plasma, EVs, and EVD fractions. Grey unfilled circles represent miRNAs with a *p*-value >0.05, and red circles represent miRNAs with a *p*-value < 0.05, and a log2FC > ±0.60 (fold change greater than 1.5).

**Figure 3:**
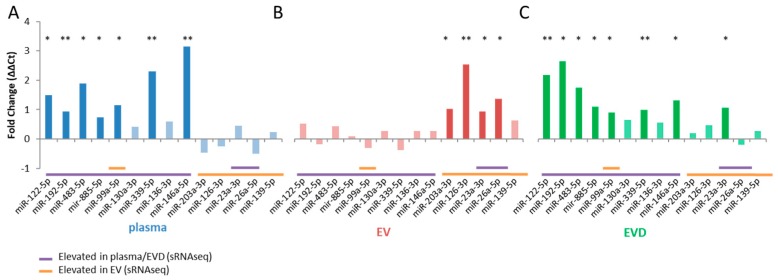
qRT-PCR validation of sRNAseq results in plasma, EVs, and EV-depleted (EVD) plasma. Log2FC for plasma (**A**), EVs (**B**) and EVD (**C**). qRT-PCR validation of sRNAseq data for selected miRNAs in plasma (**A**), EVs (**B**), and EVD fractions (**C**). Results are shown as ΔΔCt values (max cycles − (Ct reference − Ct target). MiR-451a and miR-486-5p were used as standards. Statistically significant results are designated by asterisks (* *p* < 0.05, ** *p* < 0.01). Results with *p* > 0.05 are greyed-out. Corresponding sRNAseq results are shown below as either purple bars (elevated in plasma/EVD) or yellow bars (elevated in EVs).

The miRNA spectra between control and non-metformin treated T2DM patients were analyzed to identify miRNAs associated with T2DM. Using > ±0.6 log2 fold change (~1.5 fold concentration change) and *p*-value < 0.05 as cut-off, 44 dysregulated miRNAs in whole plasma, 62 in EVs, and 108 in EV-depleted plasma were identified where the majority of them showing increased concentrations in T2DM patients (Figure 2A–C, Supplemental Table S2). Sixty eight percent of the T2DM affected miRNAs (30/44) overlap between whole plasma and EV-depleted plasma (Figure 2D). Based on the assay availability, concentration in sample, and prior reported association with T2DM, 9 miRNAs that were dysregulated in whole plasma and EV-depleted plasma (miR-99a-5p, miR-122-5p, miR-130a-3p, miR-136-3p, miR-146a-5p, miR-192-5p, miR-339-5p, miR-483-3p, miR-885-5p) and 5 dysregulated in EVs (miR-23a-3p, miR-26a-5p, miR-126-3p, miR-139-5p, miR-203a-3p) were selected for qRT-PCR verification. We were able to verify the concentration changes from 11 out of 14 selected miRNAs (7/9 from whole plasma, 8/9 from EV-depleted plasma, and 4/5 in EVs) (Figure 3A–C).

### 3.4. miRNAs Affected by Metformin Treatment

**Figure 4:**
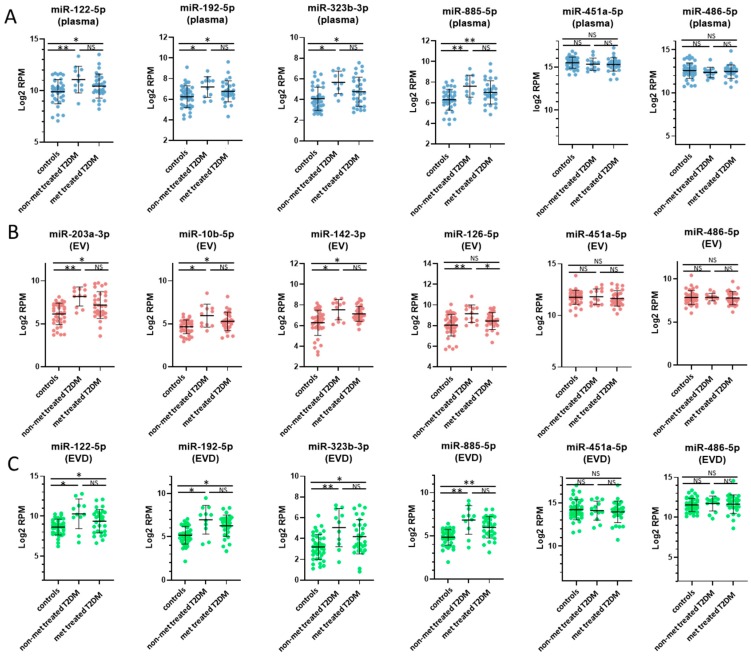
Selected miRNAs with concentration changes between controls and non-metformin (non-met) treated, and metformin-treated (met treated) T2DM. Plots of selected miRNAs showing concertation differences between controls, non-metformin treated T2DM and metformin treated T2DM patients in plasma, EV and EV-depleted samples. Plasma miRNA plots (**A**) are depicted in blue, EV miRNA plots (**B**) are depicted in red, and EV-depleted miRNA plots (**C**) are depicted in green. The values are in log2 RPM (log2 transformed reads per million), with each dot representing a patient sample. The invariant miRNAs, miR-451a-5p and miR-485-5p, are also shown. Statistically significant results are designated by asterisks (* *p* < 0.05, ** *p* < 0.01), and non-significant results are designated as ‘NS’.

When comparing controls and T2DM patients treated with metformin, 9 dysregulated miRNAs in whole plasma, 9 in EVs, and 78 in EV-depleted plasma showed concentration changes (Figure 2E–G, Supplemental Table S2). Eight out of 9 dysregulated miRNAs in whole plasma also showed concentration changes in EV-depleted plasma (Figure 2H). Compared to the miRNA identified between control and non-metformin treated T2DM patients, several affected miRNAs are in common; however, their concentration changes were smaller in metformin-treated T2DM patient samples (Supplemental Table S2) in whole plasma (Figure 4A), EVs (Figure 4B), and EV-depleted plasma (Figure 4C).

**Figure 5:**
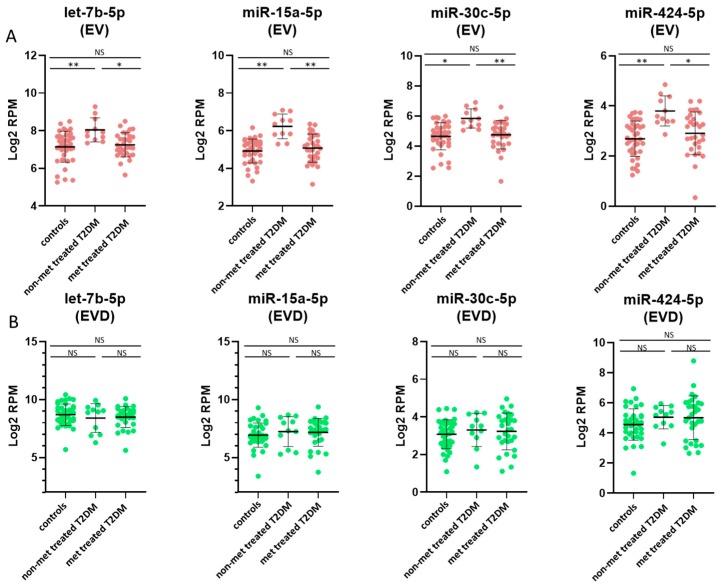
EV-specific miRNAs that reflect changes both associated with T2DM and treatment with metformin. Plots of selected miRNAs showing concertation differences between controls, non-metformin (non-met) treated T2DM and metformin (met) treated T2DM patients in EV and EV-depleted (EVD) plasma samples. EV miRNA plots (**A**) are depicted in red, and EV-depleted plasma miRNA plots (**B**) are depicted in green. The values are in log2 RPM (log2 transformed reads per million), with each dot representing a patient sample. Statistically significant results are designated by asterisks (* *p* < 0.05, ** *p* < 0.01), and non-significant results are designated as ‘NS’.

In addition to healthy controls, we also compared the miRNA profiles in whole plasma, EVs, and EV-depleted plasma between T2DM patients with or without metformin treatment. Most of the affected are from EV-encapsulated miRNA, with 42 miRNAs showed concentration differences with metformin treatment and only 2 affected miRNAs in whole plasma and none in EV-depleted plasma (Figure 2I–K). Of the 42 dysregulated miRNAs in EVs, all of them showed decreased concentrations in T2DM patients treated with metformin compared to their non-treated counterparts, (Figure 2B,J). Furthermore, many of these miRNAs also showed significant concentration changes between controls and non-metformin treated T2DM patients (Supplemental Table S2). More of the miRNAs are elevated in non-metformin treated T2DM patients, whereas metformin-treated T2DM patients have values comparable to healthy control subjects, specifically in the EV (Figure 5A), but not in the corresponding EV depleted plasma (Figure 5B).

### 3.5. Prediction of Potential Biological Pathways Associated with miRNAs

**Figure 6:**
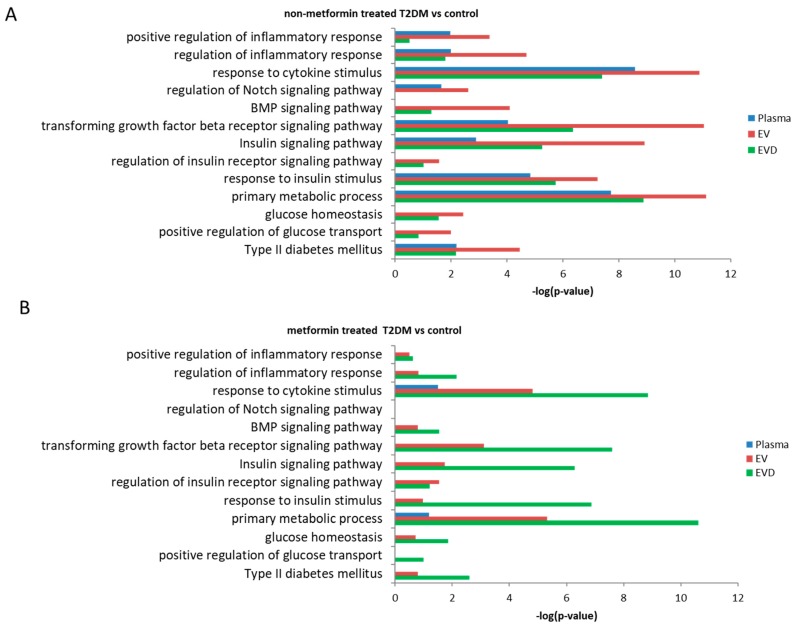
Predicted biological pathways and processes associated with T2DM in whole plasma, EVs, and EV-depleted plasma (EVD). (**A**) non-metformin (non-met) T2DM versus control, (**B**) metformin (met) treated T2DM versus control, Bar graph showing fold enrichment of selected T2DM-realted biological terms/pathways associated with plasma (blue), EV (red), and EVD (green) miRNA targets Values are –log10 transformed FDR corrected *p*-values.

We sought to identify biological processes and pathways that may be affected by miRNAs associated with T2DM in whole plasma, EVs, and EV-depleted plasma. We used the miRNAs that showed concentration changes between T2DM patients without metformin treatment and controls (Figure 2D), metformin-treated T2DM patients and controls (Figure 2H) in their respective sample types. We identified experimentally validated mRNA targets of these miRNAs using the miRTar database [27] and used the DAVID online tool [28] to identify potentially enriched biological pathways (based on KEGG terms) associated with T2DM-affected miRNAs. These pathways include regulation of homeostasis, metabolism, and uptake of glucose; signaling through insulin receptor; the TGFβ/BMP signaling pathway; the Notch signaling pathway; and regulation of the inflammatory response (Figure 6A,B). Many of these pathways were shared among whole plasma, EVs, and EV-depleted plasma, but some showed higher enrichment in EVs, such as inflammatory, insulin signaling, and glucose-related pathways.

### 3.6. Comparison of Plasma miRNAs Between Prediabetes, T2DM, and T2DM Treated with Metformin

**Figure 7:**
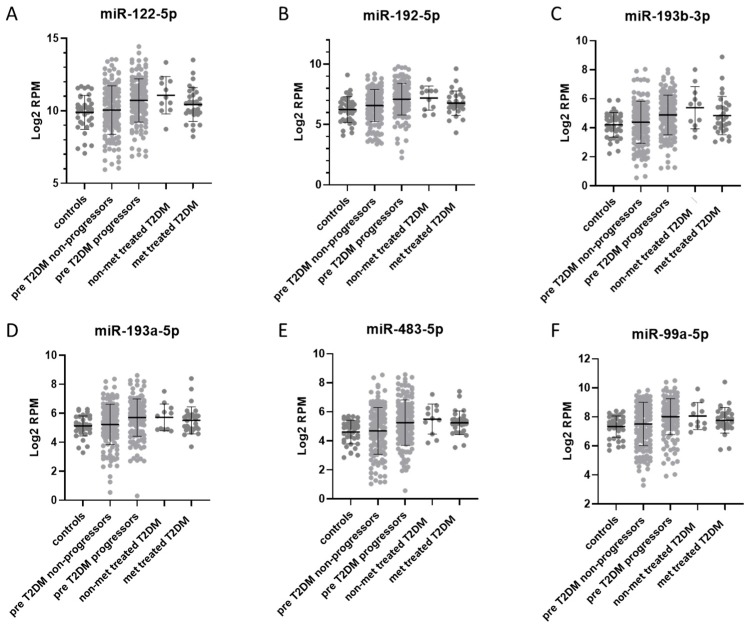
Comparison of plasma miRNAs between METSIM (prediabetic T2DM) and MetaHIT (T2DM treated with metformin). (**A**) miR-122-5p, (**B**) miR-192-5p, (**C**) miR-193b-3p, (**D**) miR-193a-5p, (**E**) miR-483-5p, (**F**) miR-99a-5p. Plots of selected plasma miRNAs that overlap between METSIM and MetaHIT datasets. The levels of miRNAs from MetaHIT data are shown as dark grey dots, while the miRNA levels from the METSIM data (Ghai et al. 2019a) are shown as light grey dots. The values are in log2 RPM (log2 transformed reads per million), with each dot representing a patient sample.

We previously used the same method to analyze plasma miRNA differences in a cohort of prediabetic individuals (METSIM study) where some progressed to T2DM and some remained at prediabetic to identify predictive marker for the onset of T2DM [20]. While the cohorts are different some of the perturbed miRNAs in plasma overlap between METSIM (the METSIM samples were at prediabetic baseline prior to T2DM progression) and MetaHIT cohorts. For example, the concentrations of miR-99a-5p, miR-122-5p, miR-192-5p, miR-193a-5p, miR-193b-5p, and miR-483-5p all were significantly changed in both between progressors and non-progressors in METSIM, and between T2DM patients (without metformin treatment) and controls in MetaHIT (Figure 7A–F). The biological processes enriched for miRNAs identified in METSIM data were also perturbed in T2DM patients in MetaHIT cohort. As with miRNA concentration changes, the enrichment of these pathways returns to near pe-diabetic (METSIM) levels in metformin-treated T2DM patients (Supplemental Figure S1).

## 4. Discussion

Circulating miRNAs may show promise as diagnostic and prognostic biomarkers for various diseases and pathologies including T2DM; however, additional studies are needed to identify and validate candidate miRNAs of clinical relevance. To understand the impact of T2DM and metformin treatment on circulating miRNA, we characterized the miRNAs in whole plasma, EVs, and EV-depleted plasma, all from subjects in the fasting state. The EV-depleted plasma contains RNA associated with RNA binding proteins such as AGO2, NPM1 [29,30] and lipoproteins—HDL and LDL complexes [25]. Since vesicle encapsulated and protein complexed RNAs arise from very different processes and may have different biological implications, analyzing the small RNA content in both EV and EV-depleted plasma gives us a richer view, in principle, of the biological status of the subjects.

In this study we identified 162 circulating miRNAs affected by T2DM in whole plasma, plasma EVs, and EV-depleted plasma. The majority of the affected miRNAs are present in whole plasma as well as EV-depleted plasma, while many T2DM-affected miRNAs in EVs were unique, suggesting EV miRNA are processed by a different mechanism than EV-depleted plasma. This observation emphasizes the usefulness of separating EV and EV-depleted biofluid in circulating RNA studies. Using qRT-PCR the concentrations changes of several of these T2DM affected miRNAs were verified. The consistency between qRT-PCR and sRNAseq results suggests that the improved EV isolation, small RNA sequencing library construction method and RNA mapping strategy provide more accurate small RNA profiles.

Many of the T2DM affected miRNAs we identified (e.g., miR-122-5p, miR-192-5p, miR-193b-5p, miR-146a-5p in plasma/EV depleted plasma; and miR-26a-5p, miR-126-5p, and miR-144-3p in EVs) have previously been shown to be dysregulated in T2DM patient plasma/serum samples (Supplementary Figure S2) [9,13,16,31]; however, this study also identified a number of new T2DM affected miRNAs, including, several T2DM affected miRNAs that were observed previously only in animal models or tissue culture (miR-136-3p, miR-382-5p, miR-495-3p in plasma [16,32] and miR-203a-3p, miR-424-5p in EVs) [33,34]. We also observed that several of these miRNAs that have been previously observed to be associated with prediabetes and T2DM (miR-122-5p, miR-192-5p, miR-193b-5p [9,13,15]) show similar concertation differences between our METSIM and MetaHIT samples sets. The higher concentration differences of these miRNAs in the MetaHIT cohort likely associated with the difference in dysregulated metabolic processes between prediabetes and T2DM. It is interesting that after been treated with metformin the levels of these miRNAs return to similar levels seen in the prediabetic individuals in the METSIM cohort (Figure 7A–F).

Due to the complex heterogenous nature of plasma and other biofluids, the exact cellular/tissue sources of these circulating miRNAs (both vesicles and non-vesicle encapsulated) are somewhat speculative. However, there are some evidences that some of these miRNAs are enriched in tissues that are associated with T2DM. For example, miR-122-5p has been shown to be expressed highly in the liver [35], and miR-126-5p, miR-146a-5p, and miR-375-3p have been shown to have some enrichment in cells of the pancreas [7]. This suggests that some of the dysregulated circulating miRNAs in T2DM may be derived from these tissues, however additional experiments are required to validate this.

In addition to the effect of T2DM, the impact of metformin treatment on the spectra of circulating miRNA was also examined. While there was no significant difference in FPG/HbA1c levels between T2DM patients in this study with or without metformin treatment, the metformin-treated T2DM patients had significantly higher circulating insulin and C-peptide levels than both non-treated T2DM patients and healthy controls (Supplementary Table S1) suggesting that these T2DM patients had a more severe form of insulin resistant T2DM. In addition, we observed that patients treated with metformin have a spectrum of circulating miRNA more similar to healthy controls in both plasma and EVs. This suggests that the metformin treatment has a “beneficial” effect based on the restoration of the circulating miRNA spectrum, though the mechanism for this effect is entirely unclear. When compared to miRNA levels in non-treated T2DM patients, the levels of some EV-encapsulated miRNAs were decreased in metformin-treated patients, suggesting a reduction in some miRNA cargo in response to metformin. For example, the concentrations of several miRNAs including let-7b-5p, miR-15a-5p, miR-15b-5p, miR-16-5p, miR-16-2-3p, miR-25-3p, miR-30b-5p, miR-106b-3p, miR-195-5p, miR-3613-5p, and miR-424-5p were increased in T2DM but decreased to the normal range after metformin treatment (Figure 5A). Unlike the miRNAs discussed above, there is little current evidence of these circulating miRNAs being involved in T2DM, aside from miR-15a-5p which has been associated with prediabetic progression to T2DM [31].

**Figure 8:**
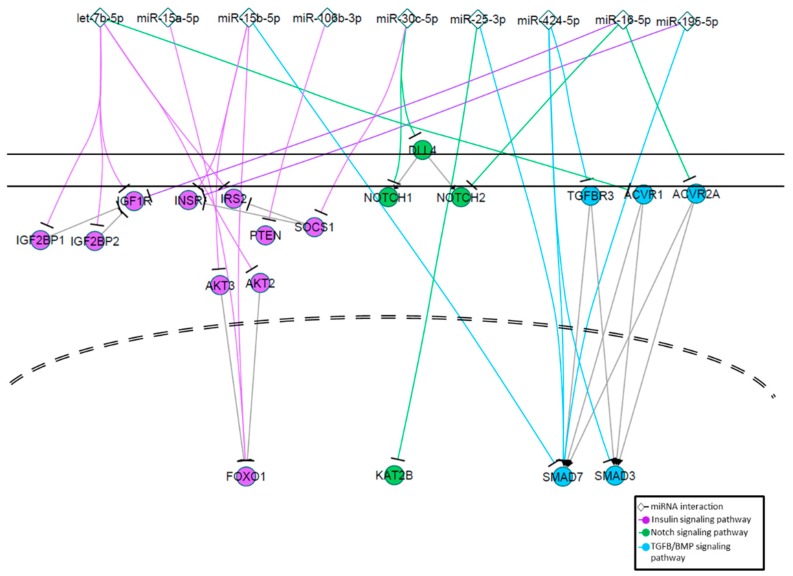
Signaling pathways modulated by EV miRNAs associated with T2DM. Pathway diagram showing selected EV miRNAs (diamonds) that change in response to metformin treatment directly regulating critical targets of insulin signaling (purple), Notch signaling (green), and TGFβ/BMP signaling (cyan).

Based on the validated gene targets of T2DM affected miRNAs in whole plasma, EVs, and EV-depleted plasma, we observed enrichment of biological pathways that are potentially associated with T2DM pathophysiology, including those related to glucose homeostasis, insulin secretion, and the TGFβ/BMP and Notch signaling pathways (Figure 8). While these pathways are predicted to be enriched (compared to controls) in non-metformin treated T2DM patients, they are not associated with patients treated with metformin. While intriguing, further studies are clearly needed to determine if these changes of biological pathways potentials relate to metformin-treatment associated changes of biochemical and physiological variables.

A few previous studies have investigated the effect of therapeutic intervention on the miRNA spectra in T2DM patients. A recent study identified 13 plasma miRNAs that decreased between T2DM patients not treated with metformin (*n* = 47) and those T2DM patients treated with metformin (*n* = 47) [36]. In our current study we also find several of these miRNAs also to be impacted by metformin. For miR-24-3p, miR-99a-5p, miR-126-5p, miR-146a-5p, and miR-152-3p in plasma, we observed a reduced fold change compared to controls between non-metformin treated T2DM and metformin treated T2DM. Additionally, we observe that miR-126-5p in EVs decreased between non-metformin treated T2DM and metformin treated T2DM. Ortega et al. examined the effect of metformin on T2DM plasma miRNAs in 12 patients (6 controls and 6 T2DM), and found that plasma miR-192-5p levels were lower in T2DM patients compared to control individuals (which should be noted is the opposite to what was observed in our current MetaHIT sample study and previous METSIM sample study). However, they found that miR-192-5p levels returned to those similar to controls in T2DM patients treated with metformin [37]. Another study of 20 T2DM patients also reported the plasma levels of miR-192-5p and miR-193b-5p return to baseline levels after 16 weeks of chronic exercise intervention [15]. While these reports studied the effect of therapeutic intervention of circulating miRNA in plasma or serum, the present study expands on previous studies to report on miRNA in whole plasma, EVs, and EV-depleted plasma, where we find the changes of miRNAs in EVs are affected by the metformin treatment compared to non-EV carriers in circulation. Therefore, miRNAs in EVs may serve as biomarkers for the response to metformin intervention in patients with T2DM.

## Figures and Tables

**Table 1:** Overview of sequencing results.

Fraction	Whole Plasma				EV				EV-Depleted Plasma (EVD)			
Sample	Control	T2DM (Non-Metformin Treated)	T2DM (Metformin Treated)	Average	Control	T2DM (Non-Metformin Treated)	T2DM (Metformin Treated)	Average	Control	T2DM (Non-Metformin Treated)	T2DM (Metformin Treated)	Average
Raw reads	9,883,520	11,530,667	10,712,888	10,709,025	9,724,030	10,252,856	9,146,014	9,707,634	10,102,865	9,629,667	10,027,784	9,920,105
Processed reads	7,103,119	7,633,060	7,373,665	7,369,948	6,747,232	6,856,590	6,254,425	6,619,416	7,911,233	7,404,532	7,579,322	7,631,695
Reads mapped to miRNA	1,156,073	1,215,783	1,166,777	1,179,544	224,714	372,528	284,460	293,901	535,018	779,273	590,431	634,907
Observed (>1 mapped)	685	706	688	693	420	445	404	423	585	642	624	617
Detected miRNAs (>10 reads)	423	472	445	446	231	273	222	242	323	383	351	352

Overview of small-RNA sequencing (sRNAseq) results, per group including for each fraction isolated. Raw reads are unfiltered reads, processed reads are after filtering for none-informative reads, reads mapped to microRNAs (miRNAs) are based on single assignment with no mismatches, Observed miRNA are miRNAs with at least one mapped read.

## Supplementary Materials

The following are available online at https://www.mdpi.com/2077-0383/8/5/617/s1, Figure S1: Fold enrichment of biological terms/pathways compared between METSIM and MetaHIT plasma miRNAs, Figure S2: Overlap between circulating miRNAs associated with T2DM and circulating miRNAs identified from MetaHIT samples, Table S1: MetaHIT patient clinical data, Table S2: miRNAs with significant concentration differences in this study.
