# Supplementary material for: Extracellular Vesicle Encapsulated MicroRNAs in Patients with Type 2 Diabetes Are Affected by Metformin Treatment
Source: J Clin Med. 2019 May 7;8(5):617. doi: 10.3390/jcm8050617 (PMC6571700; doi:10.3390/jcm8050617)
Supplement: Supplementary file 1 [file jcm-08-00617-s001.zip › jcm-499449-supppl/Ghai et al. T2DM_JCM_Suplemental.docx]

**Supplementary data**

Supplementary Table S1. MetaHIT patient clinical data

|  |  | **MetaHIT pateints (n=80)** | |  |
| --- | --- | --- | --- | --- |
|  |  | Control (n=39) | T2DM (non-metformin treated) (n=10) | T2DM (treated) (n=31) |
| **Measurements** | Age | 61.3 ± 6.1 | 63.0 ± 8.7 | 61.7 ± 5.8 |
|  | BMI | 27.9 ± 5.0 | 24.2 ± 5.4 | 28.9 ± 5.5 |
|  | FPG (mmol/L) | 5.53 ± 0.33 | 7.64 ± 2.05 | 8.10 ± 1.89 |
|  | HbA1c (%) | 5.46 ± 0.20 | 6.88 ± 1.19 | 7.30 ± 1.28 |
|  | Insulin (pmol/L) | 43.77 ± 25.04 | 36.67 ± 10.11 | 61.06 ± 43.57 |
|  | C-peptide (pmol/L) | 680.43 ± 337.19 | 566.90 ± 278.45 | 873.129 ± 493.771 |
|  | HOMA-IR | 1.57 ± 0.92 | 2.35 ± 0.69 | 3.41 ± 3.33 |
|  | M,F | 16,23 | 6,4 | 12,19 |
|  |  | control: T2DM (non-treated) | control: T2DM (treated) | T2DM (non-treated): T2DM (treated) |
| **P-value** | Age | 0.57157 | 0.77942 | 0.66709 |
|  | BMI | 0.07232 | 0.45597 | 0.20281 |
|  | FPG | 0.00993 | 1.76E-08 | 0.53981 |
|  | HbA1c | 0.00471 | 6.89E-09 | 0.36166 |
|  | Insulin | 0.18438 | 0.05536 | 0.00684 |
|  | C-peptide | 0.29882 | 0.06927 | 0.02298 |
|  | HOMA-IR | 0.00851 | 0.00511 | 0.00684 |

Supplemental Table S2. miRNAs with significant concentration differences in this stud

|  | control/T2DM (non-metfromin treated) | | | | | | control/T2DM (treated) | | | | | | T2DM (non-metformin treated)/T2DM (treated) | | | | | |
| --- | --- | --- | --- | --- | --- | --- | --- | --- | --- | --- | --- | --- | --- | --- | --- | --- | --- | --- |
|  | **plasma** | | **EVs** | | **EVD** | | **plasma** | | **EVs** | | **EVD** | | **plasma** | | **EVs** | | **EVD** | |
| miRNA ID | **log2FC** | **p-value** | **log2FC** | **p-value** | **log2FC** | **p-value** | **log2FC** | **p-value** | **log2FC** | **p-value** | **log2FC** | **p-value** | **log2FC** | **p-value** | **log2FC** | **p-value** | **log2FC** | **p-value** |
| let-7a-1-5p | ─ | ─ | 0.87 | 0.00306 | ─ | ─ | ─ | ─ | ─ | ─ | ─ | ─ | ─ | ─ | ─ | ─ | ─ | ─ |
| let-7b-5p | ─ | ─ | 0.76 | 0.00602 | ─ | ─ | ─ | ─ | ─ | ─ | ─ | ─ | ─ | ─ | -0.62 | 0.01890 | ─ | ─ |
| let-7c-5p | ─ | ─ | 0.94 | 0.02508 | ─ | ─ | ─ | ─ | ─ | ─ | ─ | ─ | ─ | ─ | ─ | ─ | ─ | ─ |
| let-7d-3p | ─ | ─ | ─ | ─ | 1.14 | 0.029285 | ─ | ─ | ─ | ─ | 0.73 | 0.00361 | ─ | ─ | ─ | ─ | ─ | ─ |
| let-7d-5p | ─ | ─ | 0.67 | 0.02722 | ─ | ─ | ─ | ─ | ─ | ─ | ─ | ─ | ─ | ─ | ─ | ─ | ─ | ─ |
| let-7e-5p | ─ | ─ | 1.02 | 0.01941 | ─ | ─ | ─ | ─ | ─ | ─ | 0.63 | 0.01141 | ─ | ─ | ─ | ─ | ─ | ─ |
| let-7f-1-5p | ─ | ─ | 0.77 | 0.01908 | ─ | ─ | ─ | ─ | ─ | ─ | ─ | ─ | ─ | ─ | ─ | ─ | ─ | ─ |
| let-7f-2-5p | ─ | ─ | ─ | ─ | ─ | ─ | ─ | ─ | 0.74 | 0.00609 | ─ | ─ | ─ | ─ | ─ | ─ | ─ | ─ |
| let-7g-5p | ─ | ─ | 0.78 | 0.01302 | ─ | ─ | ─ | ─ | ─ | ─ | ─ | ─ | ─ | ─ | ─ | ─ | ─ | ─ |
| let-7i-5p | ─ | ─ | 1.07 | 0.00167 | ─ | ─ | ─ | ─ | ─ | ─ | ─ | ─ | ─ | ─ | ─ | ─ | ─ | ─ |
| miR-100-5p | ─ | ─ | ─ | ─ | 1.31 | 0.005247 | ─ | ─ | ─ | ─ | 1.04 | 0.00021 | ─ | ─ | ─ | ─ | ─ | ─ |
| miR-101-1-3p | ─ | ─ | 0.73 | 0.03356 | ─ | ─ | ─ | ─ | ─ | ─ | ─ | ─ | ─ | ─ | -0.71 | 0.03656 | ─ | ─ |
| miR-103a-1-3p | ─ | ─ | 0.80 | 0.00822 | ─ | ─ | ─ | ─ | ─ | ─ | ─ | ─ | ─ | ─ | ─ | ─ | ─ | ─ |
| miR-106b-3p | ─ | ─ | 0.90 | 0.00952 | ─ | ─ | ─ | ─ | ─ | ─ | ─ | ─ | ─ | ─ | -0.68 | 0.04191 | ─ | ─ |
| miR-10a-5p | ─ | ─ | 0.95 | 0.00991 | 1.28 | 0.016647 | ─ | ─ | ─ | ─ | 0.91 | 0.00204 | ─ | ─ | -0.79 | 0.02516 | ─ | ─ |
| miR-10b-5p | ─ | ─ | 1.38 | 0.01951 | 1.10 | 0.037773 | ─ | ─ | 0.66 | 0.02095 | 0.73 | 0.01576 | ─ | ─ | ─ | ─ | ─ | ─ |
| miR-122-5p | 0.86 | 0.00610 | ─ | ─ | 1.53 | 0.024645 | 0.65 | 0.02714 | ─ | ─ | 0.71 | 0.01572 | ─ | ─ | -0.71 | 0.02969 | ─ | ─ |
| miR-1246-5p | ─ | ─ | ─ | ─ | ─ | ─ | ─ | ─ | ─ | ─ | ─ | ─ | ─ | ─ | -0.95 | 0.03088 | ─ | ─ |
| miR-125a-5p | ─ | ─ | 0.98 | 0.00473 | 0.81 | 0.043992 | ─ | ─ | ─ | ─ | ─ | ─ | ─ | ─ | -0.86 | 0.01126 | ─ | ─ |
| miR-125b-1-5p | ─ | ─ | 0.83 | 0.00982 | ─ | ─ | ─ | ─ | ─ | ─ | 0.67 | 0.00523 | ─ | ─ | -0.82 | 0.01148 | ─ | ─ |
| miR-126-3p | ─ | ─ | 0.93 | 0.00875 | ─ | ─ | ─ | ─ | ─ | ─ | ─ | ─ | ─ | ─ | ─ | ─ | ─ | ─ |
| miR-126-5p | 0.68 | 0.00072 | 1.07 | 0.00189 | 1.15 | 0.048493 | ─ | ─ | ─ | ─ | 0.65 | 0.02940 | ─ | ─ | -0.63 | 0.04808 | ─ | ─ |
| miR-128-1-3p | 0.64 | 0.00311 | ─ | ─ | 1.20 | 0.026846 | ─ | ─ | ─ | ─ | 0.74 | 0.00558 | ─ | ─ | ─ | ─ | ─ | ─ |
| miR-1306-5p | ─ | ─ | ─ | ─ | 0.90 | 0.028672 | ─ | ─ | ─ | ─ | 0.69 | 0.00111 | ─ | ─ | ─ | ─ | ─ | ─ |
| miR-1307-5p | ─ | ─ | ─ | ─ | 1.30 | 0.010481 | ─ | ─ | ─ | ─ | ─ | ─ | ─ | ─ | ─ | ─ | ─ | ─ |
| miR-130a-3p | 0.76 | 0.00258 | ─ | ─ | 1.15 | 0.034798 | ─ | ─ | ─ | ─ | ─ | ─ | ─ | ─ | -0.66 | 0.04235 | ─ | ─ |
| miR-130b-3p | 0.60 | 0.00214 | ─ | ─ | 1.05 | 0.023719 | ─ | ─ | ─ | ─ | 0.65 | 0.00891 | ─ | ─ | ─ | ─ | ─ | ─ |
| miR-130b-5p | ─ | ─ | ─ | ─ | 0.89 | 0.036985 | ─ | ─ | ─ | ─ | 0.80 | 0.00505 | ─ | ─ | ─ | ─ | ─ | ─ |
| miR-132-3p | ─ | ─ | ─ | ─ | 1.10 | 0.001756 | ─ | ─ | ─ | ─ | 0.72 | 0.00151 | ─ | ─ | -1.28 | 0.03288 | ─ | ─ |
| miR-134-5p | 1.12 | 0.02299 | ─ | ─ | 1.29 | 0.044103 | ─ | ─ | ─ | ─ | 0.71 | 0.04305 | ─ | ─ | ─ | ─ | ─ | ─ |
| miR-136-3p | ─ | ─ | ─ | ─ | 1.18 | 0.047822 | ─ | ─ | ─ | ─ | 0.86 | 0.01047 | -0.69 | 0.048244 | ─ | ─ | ─ | ─ |
| miR-139-5p | ─ | ─ | 1.04 | 0.00613 | ─ | ─ | ─ | ─ | ─ | ─ | ─ | ─ | ─ | ─ | ─ | ─ | ─ | ─ |
| miR-140-3p | ─ | ─ | ─ | ─ | 0.77 | 0.049502 | ─ | ─ | ─ | ─ | ─ | ─ | ─ | ─ | ─ | ─ | ─ | ─ |
| miR-141-3p | ─ | ─ | ─ | ─ | ─ | ─ | ─ | ─ | ─ | ─ | 0.84 | 0.00541 | ─ | ─ | ─ | ─ | ─ | ─ |
| miR-142-3p | ─ | ─ | 1.13 | 0.00255 | ─ | ─ | ─ | ─ | 0.90 | 0.00039 | ─ | ─ | ─ | ─ | ─ | ─ | ─ | ─ |
| miR-142-5p | ─ | ─ | ─ | ─ | 1.14 | 0.019542 | ─ | ─ | ─ | ─ | 0.69 | 0.01263 | ─ | ─ | ─ | ─ | ─ | ─ |
| miR-144-3p | ─ | ─ | 0.77 | 0.03799 | ─ | ─ | ─ | ─ | ─ | ─ | ─ | ─ | ─ | ─ | ─ | ─ | ─ | ─ |
| miR-145-5p | ─ | ─ | ─ | ─ | 0.75 | 0.019299 | ─ | ─ | ─ | ─ | ─ | ─ | ─ | ─ | -0.75 | 0.03061 | ─ | ─ |
| miR-146a-5p | 0.72 | 0.00578 | ─ | ─ | 1.24 | 0.01417 | ─ | ─ | ─ | ─ | 0.64 | 0.02150 | ─ | ─ | ─ | ─ | ─ | ─ |
| miR-146b-5p | ─ | ─ | ─ | ─ | 0.94 | 0.026778 | ─ | ─ | ─ | ─ | ─ | ─ | ─ | ─ | ─ | ─ | ─ | ─ |
| miR-148a-3p | ─ | ─ | ─ | ─ | 1.47 | 0.005268 | ─ | ─ | ─ | ─ | 0.82 | 0.00107 | ─ | ─ | -0.77 | 0.04192 | ─ | ─ |
| miR-148b-3p | ─ | ─ | 0.84 | 0.01978 | 1.20 | 0.018956 | ─ | ─ | ─ | ─ | 0.70 | 0.00956 | ─ | ─ | -0.97 | 0.01007 | ─ | ─ |
| miR-150-5p | ─ | ─ | 0.71 | 0.03781 | ─ | ─ | ─ | ─ | ─ | ─ | ─ | ─ | ─ | ─ | ─ | ─ | ─ | ─ |
| miR-151a-3p | 0.90 | 0.00287 | ─ | ─ | 1.39 | 0.013572 | ─ | ─ | ─ | ─ | 0.74 | 0.00968 | ─ | ─ | ─ | ─ | ─ | ─ |
| miR-151a-5p | 0.62 | 0.00283 | 0.75 | 0.04224 | 1.13 | 0.02804 | ─ | ─ | ─ | ─ | ─ | ─ | ─ | ─ | ─ | ─ | ─ | ─ |
| miR-152-3p | 0.79 | 0.00336 | ─ | ─ | 1.51 | 0.002214 | ─ | ─ | ─ | ─ | 0.76 | 0.00462 | ─ | ─ | ─ | ─ | ─ | ─ |
| miR-155-5p | ─ | ─ | 1.05 | 0.01913 | 0.68 | 0.036173 | ─ | ─ | 0.70 | 0.00158 | ─ | ─ | ─ | ─ | ─ | ─ | ─ | ─ |
| miR-15a-5p | ─ | ─ | 0.87 | 0.00657 | ─ | ─ | ─ | ─ | ─ | ─ | ─ | ─ | ─ | ─ | -0.90 | 0.00441 | ─ | ─ |
| miR-15b-5p | ─ | ─ | 0.77 | 0.01128 | ─ | ─ | ─ | ─ | ─ | ─ | ─ | ─ | ─ | ─ | -0.76 | 0.01812 | ─ | ─ |
| miR-16-1-5p | ─ | ─ | 0.67 | 0.04516 | ─ | ─ | ─ | ─ | ─ | ─ | ─ | ─ | ─ | ─ | -0.81 | 0.01457 | ─ | ─ |
| miR-16-2-3p | ─ | ─ | 0.96 | 0.00954 | ─ | ─ | ─ | ─ | ─ | ─ | ─ | ─ | ─ | ─ | -0.72 | 0.03787 | ─ | ─ |
| miR-181a-1-5p | ─ | ─ | 0.86 | 0.01839 | 1.09 | 0.010687 | ─ | ─ | ─ | ─ | 0.68 | 0.00628 | ─ | ─ | ─ | ─ | ─ | ─ |
| miR-181b-1-5p | ─ | ─ | ─ | ─ | 0.91 | 0.034325 | ─ | ─ | ─ | ─ | 0.62 | 0.01063 | ─ | ─ | ─ | ─ | ─ | ─ |
| miR-181c-5p | ─ | ─ | 0.65 | 0.02923 | 0.82 | 0.047865 | ─ | ─ | ─ | ─ | ─ | ─ | ─ | ─ | ─ | ─ | ─ | ─ |
| miR-183-5p | ─ | ─ | ─ | ─ | ─ | ─ | ─ | ─ | ─ | ─ | ─ | ─ | ─ | ─ | -1.29 | 0.02096 | ─ | ─ |
| miR-186-5p | ─ | ─ | 0.80 | 0.03269 | 0.90 | 0.046193 | ─ | ─ | ─ | ─ | ─ | ─ | ─ | ─ | ─ | ─ | ─ | ─ |
| miR-191-5p | ─ | ─ | 0.64 | 0.02712 | ─ | ─ | ─ | ─ | ─ | ─ | ─ | ─ | ─ | ─ | ─ | ─ | ─ | ─ |
| miR-192-5p | 0.72 | 0.03919 | ─ | ─ | 1.82 | 0.00195 | 0.61 | 0.01777 | ─ | ─ | 1.04 | 0.00037 | ─ | ─ | ─ | ─ | ─ | ─ |
| miR-193a-5p | 0.61 | 0.00722 | ─ | ─ | ─ | ─ | ─ | ─ | ─ | ─ | 0.60 | 0.00420 | ─ | ─ | -0.95 | 0.04150 | ─ | ─ |
| miR-193b-3p | 0.81 | 0.00874 | ─ | ─ | 1.24 | 0.047601 | 0.76 | 0.00874 | ─ | ─ | 0.65 | 0.01775 | ─ | ─ | ─ | ─ | ─ | ─ |
| miR-194-1-5p | ─ | ─ | ─ | ─ | 1.56 | 0.00646 | ─ | ─ | ─ | ─ | 0.92 | 0.00176 | ─ | ─ | ─ | ─ | ─ | ─ |
| miR-195-5p | ─ | ─ | 0.93 | 0.01346 | ─ | ─ | ─ | ─ | ─ | ─ | ─ | ─ | ─ | ─ | -0.73 | 0.03888 | ─ | ─ |
| miR-197-3p | 0.67 | 0.00284 | ─ | ─ | ─ | ─ | ─ | ─ | ─ | ─ | ─ | ─ | ─ | ─ | ─ | ─ | ─ | ─ |
| miR-199a-1-3p | 0.66 | 0.01153 | ─ | ─ | 1.12 | 0.045481 | ─ | ─ | ─ | ─ | ─ | ─ | ─ | ─ | ─ | ─ | ─ | ─ |
| miR-200a-3p | ─ | ─ | ─ | ─ | 1.00 | 0.019555 | ─ | ─ | ─ | ─ | 0.76 | 0.00229 | ─ | ─ | ─ | ─ | ─ | ─ |
| miR-200b-3p | ─ | ─ | ─ | ─ | 1.06 | 0.011764 | ─ | ─ | ─ | ─ | 0.84 | 0.00037 | ─ | ─ | ─ | ─ | ─ | ─ |
| miR-200c-3p | ─ | ─ | ─ | ─ | ─ | ─ | ─ | ─ | ─ | ─ | 0.78 | 0.01422 | ─ | ─ | ─ | ─ | ─ | ─ |
| miR-203a-3p | -0.94 | 0.02171 | 1.57 | 0.00134 | ─ | ─ | ─ | ─ | 0.91 | 0.02160 | ─ | ─ | ─ | ─ | ─ | ─ | ─ | ─ |
| miR-205-5p | ─ | ─ | ─ | ─ | ─ | ─ | ─ | ─ | ─ | ─ | ─ | ─ | ─ | ─ | -1.19 | 0.01631 | ─ | ─ |
| miR-20b-5p | ─ | ─ | ─ | ─ | ─ | ─ | ─ | ─ | -0.69 | 0.00140 | ─ | ─ | ─ | ─ | -0.78 | 0.02431 | ─ | ─ |
| miR-210-3p | ─ | ─ | ─ | ─ | 1.07 | 0.013314 | ─ | ─ | ─ | ─ | 0.61 | 0.01320 | ─ | ─ | ─ | ─ | ─ | ─ |
| miR-21-3p | ─ | ─ | ─ | ─ | 1.16 | 0.01558 | ─ | ─ | ─ | ─ | ─ | ─ | ─ | ─ | ─ | ─ | ─ | ─ |
| miR-214-3p | ─ | ─ | ─ | ─ | 0.65 | 0.011753 | ─ | ─ | ─ | ─ | ─ | ─ | ─ | ─ | ─ | ─ | ─ | ─ |
| miR-21-5p | ─ | ─ | 0.67 | 0.03890 | 1.37 | 0.010694 | ─ | ─ | ─ | ─ | 0.87 | 0.00065 | ─ | ─ | ─ | ─ | ─ | ─ |
| miR-221-3p | 0.70 | 0.00332 | 0.71 | 0.00828 | 1.07 | 0.026021 | ─ | ─ | ─ | ─ | ─ | ─ | ─ | ─ | -0.64 | 0.01396 | ─ | ─ |
| miR-222-3p | ─ | ─ | 0.72 | 0.04406 | 0.90 | 0.039089 | ─ | ─ | ─ | ─ | ─ | ─ | ─ | ─ | ─ | ─ | ─ | ─ |
| miR-223-3p | 0.77 | 0.00315 | ─ | ─ | ─ | ─ | ─ | ─ | ─ | ─ | ─ | ─ | ─ | ─ | ─ | ─ | ─ | ─ |
| miR-223-5p | ─ | ─ | ─ | ─ | 0.87 | 0.028287 | ─ | ─ | ─ | ─ | ─ | ─ | ─ | ─ | -0.99 | 0.01920 | ─ | ─ |
| miR-22-3p | ─ | ─ | 0.64 | 0.02938 | 1.08 | 0.035405 | ─ | ─ | ─ | ─ | ─ | ─ | ─ | ─ | -0.75 | 0.01012 | ─ | ─ |
| miR-224-5p | ─ | ─ | 0.81 | 0.03996 | ─ | ─ | ─ | ─ | ─ | ─ | 0.69 | 0.01044 | ─ | ─ | ─ | ─ | ─ | ─ |
| miR-22-5p | ─ | ─ | ─ | ─ | 1.15 | 0.032455 | ─ | ─ | ─ | ─ | ─ | ─ | ─ | ─ | -0.83 | 0.00359 | ─ | ─ |
| miR-2355-3p | ─ | ─ | ─ | ─ | 0.92 | 0.045373 | ─ | ─ | ─ | ─ | 0.62 | 0.03352 | ─ | ─ | ─ | ─ | ─ | ─ |
| miR-23a-3p | ─ | ─ | 0.80 | 0.00372 | 1.12 | 0.022067 | ─ | ─ | ─ | ─ | 0.66 | 0.01306 | ─ | ─ | -0.63 | 0.01704 | ─ | ─ |
| miR-23b-3p | ─ | ─ | 0.93 | 0.01002 | 1.35 | 0.004485 | ─ | ─ | ─ | ─ | 0.97 | 0.00031 | ─ | ─ | ─ | ─ | ─ | ─ |
| miR-24-1-3p | 0.65 | 0.00299 | ─ | ─ | 1.23 | 0.020167 | ─ | ─ | ─ | ─ | 0.62 | 0.02385 | ─ | ─ | ─ | ─ | ─ | ─ |
| miR-24-2-5p | ─ | ─ | ─ | ─ | 0.75 | 0.033244 | ─ | ─ | ─ | ─ | ─ | ─ | ─ | ─ | ─ | ─ | ─ | ─ |
| miR-25-3p | ─ | ─ | 0.85 | 0.03142 | ─ | ─ | ─ | ─ | ─ | ─ | ─ | ─ | ─ | ─ | -0.88 | 0.02423 | ─ | ─ |
| miR-26a-1-5p | ─ | ─ | 0.93 | 0.00514 | 0.89 | 0.017114 | ─ | ─ | ─ | ─ | 0.66 | 0.00404 | ─ | ─ | ─ | ─ | ─ | ─ |
| miR-26b-5p | ─ | ─ | 0.92 | 0.00301 | ─ | ─ | ─ | ─ | ─ | ─ | ─ | ─ | ─ | ─ | ─ | ─ | ─ | ─ |
| miR-27a-3p | ─ | ─ | 1.06 | 0.00217 | 1.17 | 0.036161 | ─ | ─ | ─ | ─ | 0.82 | 0.00596 | ─ | ─ | ─ | ─ | ─ | ─ |
| miR-27b-3p | 0.82 | 0.00044 | 1.02 | 0.00245 | 1.78 | 0.00587 | ─ | ─ | ─ | ─ | 1.03 | 0.00114 | ─ | ─ | -0.83 | 0.00972 | ─ | ─ |
| miR-28-3p | 0.70 | 0.00689 | 1.01 | 0.01240 | 1.25 | 0.01633 | ─ | ─ | ─ | ─ | 0.73 | 0.00176 | ─ | ─ | ─ | ─ | ─ | ─ |
| miR-28-5p | ─ | ─ | ─ | ─ | 1.39 | 0.003877 | ─ | ─ | ─ | ─ | 0.75 | 0.00481 | ─ | ─ | ─ | ─ | ─ | ─ |
| miR-29a-3p | ─ | ─ | 0.69 | 0.02783 | 1.30 | 0.005207 | ─ | ─ | ─ | ─ | 0.85 | 0.00105 | ─ | ─ | ─ | ─ | ─ | ─ |
| miR-29c-3p | ─ | ─ | ─ | ─ | 1.09 | 0.019363 | ─ | ─ | ─ | ─ | ─ | ─ | ─ | ─ | ─ | ─ | ─ | ─ |
| miR-30a-3p | 1.04 | 3.1E-05 | 0.98 | 0.00768 | 0.96 | 0.018826 | ─ | ─ | ─ | ─ | 0.64 | 0.00048 | ─ | ─ | ─ | ─ | ─ | ─ |
| miR-30a-5p | ─ | ─ | 0.81 | 0.02030 | 1.35 | 0.003072 | ─ | ─ | ─ | ─ | 0.73 | 0.00318 | ─ | ─ | -0.78 | 0.02206 | ─ | ─ |
| miR-30b-5p | ─ | ─ | 0.66 | 0.04861 | ─ | ─ | ─ | ─ | ─ | ─ | 0.64 | 0.01039 | ─ | ─ | ─ | ─ | ─ | ─ |
| miR-30c-1-5p | ─ | ─ | 0.70 | 0.01975 | ─ | ─ | ─ | ─ | ─ | ─ | ─ | ─ | ─ | ─ | -0.62 | 0.00754 | ─ | ─ |
| miR-30d-5p | ─ | ─ | ─ | ─ | 1.10 | 0.020787 | ─ | ─ | ─ | ─ | ─ | ─ | ─ | ─ | -0.63 | 0.04585 | ─ | ─ |
| miR-30e-3p | ─ | ─ | 1.26 | 0.00773 | ─ | ─ | ─ | ─ | ─ | ─ | ─ | ─ | ─ | ─ | ─ | ─ | ─ | ─ |
| miR-30e-5p | ─ | ─ | ─ | ─ | 1.05 | 0.022656 | ─ | ─ | ─ | ─ | ─ | ─ | ─ | ─ | ─ | ─ | ─ | ─ |
| miR-320a-3p | ─ | ─ | ─ | ─ | ─ | ─ | ─ | ─ | ─ | ─ | ─ | ─ | ─ | ─ | -0.72 | 0.01695 | ─ | ─ |
| miR-323a-3p | 1.25 | 0.00437 | ─ | ─ | 1.32 | 0.023298 | ─ | ─ | ─ | ─ | 0.86 | 0.01388 | ─ | ─ | ─ | ─ | ─ | ─ |
| miR-323b-3p | 1.53 | 0.00113 | ─ | ─ | 1.50 | 0.020503 | 0.67 | 0.027437 | ─ | ─ | 1.09 | 0.00400 | -0.86 | 0.042698 | ─ | ─ | ─ | ─ |
| miR-32-5p | ─ | ─ | ─ | ─ | 0.87 | 0.037342 | ─ | ─ | ─ | ─ | 0.65 | 0.02020 | ─ | ─ | ─ | ─ | ─ | ─ |
| miR-328-3p | ─ | ─ | ─ | ─ | 1.20 | 0.011782 | ─ | ─ | ─ | ─ | ─ | ─ | ─ | ─ | ─ | ─ | ─ | ─ |
| miR-329-1-3p | 0.91 | 0.01088 | ─ | ─ | ─ | ─ | ─ | ─ | ─ | ─ | ─ | ─ | ─ | ─ | ─ | ─ | ─ | ─ |
| miR-330-3p | 0.64 | 0.04037 | ─ | ─ | 1.09 | 0.014186 | ─ | ─ | ─ | ─ | ─ | ─ | ─ | ─ | ─ | ─ | ─ | ─ |
| miR-335-5p | 0.67 | 0.00844 | ─ | ─ | 1.33 | 0.026794 | ─ | ─ | ─ | ─ | 0.87 | 0.00744 | ─ | ─ | ─ | ─ | ─ | ─ |
| miR-338-5p | ─ | ─ | ─ | ─ | 0.88 | 0.032831 | ─ | ─ | ─ | ─ | 0.76 | 0.00493 | ─ | ─ | ─ | ─ | ─ | ─ |
| miR-339-3p | 0.67 | 0.0016 | ─ | ─ | ─ | ─ | ─ | ─ | ─ | ─ | ─ | ─ | ─ | ─ | ─ | ─ | ─ | ─ |
| miR-339-5p | 0.82 | 0.00189 | ─ | ─ | 1.00 | 0.024428 | ─ | ─ | ─ | ─ | ─ | ─ | ─ | ─ | ─ | ─ | ─ | ─ |
| miR-340-5p | ─ | ─ | ─ | ─ | 1.12 | 0.037027 | ─ | ─ | ─ | ─ | ─ | ─ | ─ | ─ | ─ | ─ | ─ | ─ |
| miR-342-3p | ─ | ─ | ─ | ─ | 1.24 | 0.005688 | ─ | ─ | ─ | ─ | 0.69 | 0.00298 | ─ | ─ | ─ | ─ | ─ | ─ |
| miR-345-5p | ─ | ─ | ─ | ─ | 1.00 | 0.015629 | ─ | ─ | ─ | ─ | ─ | ─ | ─ | ─ | -1.05 | 0.03412 | ─ | ─ |
| miR-34a-5p | ─ | ─ | ─ | ─ | 1.38 | 0.007661 | ─ | ─ | ─ | ─ | 0.76 | 0.00174 | ─ | ─ | -0.81 | 0.02791 | ─ | ─ |
| miR-3613-5p | ─ | ─ | 1.087454 | 0.000489 | ─ | ─ | ─ | ─ | ─ | ─ | ─ | ─ | ─ | ─ | -0.80 | 0.00629 | ─ | ─ |
| miR-361-5p | ─ | ─ | ─ | ─ | 1.38 | 0.009769 | ─ | ─ | ─ | ─ | 0.84 | 0.00232 | ─ | ─ | ─ | ─ | ─ | ─ |
| miR-362-3p | ─ | ─ | ─ | ─ | 0.79 | 0.040269 | ─ | ─ | ─ | ─ | ─ | ─ | ─ | ─ | ─ | ─ | ─ | ─ |
| miR-365a-3p | ─ | ─ | ─ | ─ | 1.56 | 0.01473 | ─ | ─ | ─ | ─ | 0.74 | 0.00524 | ─ | ─ | ─ | ─ | ─ | ─ |
| miR-369-3p | 1.30 | 0.00945 | ─ | ─ | 1.54 | 0.03711 | ─ | ─ | ─ | ─ | 0.95 | 0.01717 | ─ | ─ | ─ | ─ | ─ | ─ |
| miR-374a-5p | ─ | ─ | 0.90 | 0.01749 | 0.85 | 0.020988 | ─ | ─ | ─ | ─ | 0.81 | 0.00054 | ─ | ─ | ─ | ─ | ─ | ─ |
| miR-374b-5p | ─ | ─ | ─ | ─ | ─ | ─ | ─ | ─ | ─ | ─ | 0.64 | 0.01351 | ─ | ─ | ─ | ─ | ─ | ─ |
| miR-375-3p | ─ | ─ | ─ | ─ | 1.23 | 0.029279 | 0.82 | 0.00303 | -0.61 | 0.02139 | 1.21 | 0.00014 | ─ | ─ | ─ | ─ | ─ | ─ |
| miR-376c-3p | 0.91 | 0.01057 | ─ | ─ | 1.36 | 0.044505 | ─ | ─ | ─ | ─ | ─ | ─ | ─ | ─ | ─ | ─ | ─ | ─ |
| miR-378a-3p | ─ | ─ | ─ | ─ | 1.25 | 0.004547 | ─ | ─ | ─ | ─ | 0.70 | 0.00453 | ─ | ─ | -1.10 | 0.04636 | ─ | ─ |
| miR-382-5p | 1.34 | 0.00349 | ─ | ─ | 1.34 | 0.04464 | ─ | ─ | ─ | ─ | 0.71 | 0.04744 | ─ | ─ | ─ | ─ | ─ | ─ |
| miR-409-3p | 1.14 | 0.02879 | ─ | ─ | ─ | ─ | ─ | ─ | ─ | ─ | 0.77 | 0.02327 | ─ | ─ | ─ | ─ | ─ | ─ |
| miR-423-3p | ─ | ─ | ─ | ─ | 1.05 | 0.02561 | ─ | ─ | ─ | ─ | ─ | ─ | ─ | ─ | ─ | ─ | ─ | ─ |
| miR-424-3p | ─ | ─ | ─ | ─ | ─ | ─ | ─ | ─ | ─ | ─ | 0.80 | 0.00273 | ─ | ─ | ─ | ─ | ─ | ─ |
| miR-424-5p | ─ | ─ | 0.96 | 0.00614 | ─ | ─ | ─ | ─ | ─ | ─ | ─ | ─ | ─ | ─ | -0.76 | 0.02707 | ─ | ─ |
| miR-4286-5p | 1.07 | 0.00171 | ─ | ─ | 1.27 | 0.04217 | 0.63 | 0.02024 | ─ | ─ | 0.67 | 0.03269 | ─ | ─ | ─ | ─ | ─ | ─ |
| miR-432-5p | 1.48 | 0.00432 | ─ | ─ | 1.25 | 0.045617 | ─ | ─ | ─ | ─ | ─ | ─ | ─ | ─ | ─ | ─ | ─ | ─ |
| miR-4532-5p | ─ | ─ | ─ | ─ | ─ | ─ | ─ | ─ | -0.81 | 0.00014 | ─ | ─ | ─ | ─ | -0.97 | 0.00054 | ─ | ─ |
| miR-483-3p | ─ | ─ | ─ | ─ | 1.01 | 0.018243 | ─ | ─ | ─ | ─ | 0.70 | 0.00353 | ─ | ─ | ─ | ─ | ─ | ─ |
| miR-483-5p | 0.75 | 0.04785 | ─ | ─ | 1.22 | 0.026589 | 0.68 | 0.000922 | ─ | ─ | 0.79 | 0.00125 | ─ | ─ | ─ | ─ | ─ | ─ |
| miR-487b-3p | 1.06 | 0.01139 | ─ | ─ | 1.37 | 0.034089 | ─ | ─ | ─ | ─ | ─ | ─ | ─ | ─ | ─ | ─ | ─ | ─ |
| miR-494-3p | ─ | ─ | ─ | ─ | 1.09 | 0.023613 | ─ | ─ | ─ | ─ | ─ | ─ | ─ | ─ | ─ | ─ | ─ | ─ |
| miR-495-3p | 0.97 | 0.01179 | ─ | ─ | 1.16 | 0.0361 | ─ | ─ | 0.61 | 0.02456 | 0.78 | 0.01535 | ─ | ─ | ─ | ─ | ─ | ─ |
| miR-500a-3p | ─ | ─ | ─ | ─ | 0.74 | 0.04451 | ─ | ─ | ─ | ─ | ─ | ─ | ─ | ─ | ─ | ─ | ─ | ─ |
| miR-502-3p | ─ | ─ | ─ | ─ | 0.75 | 0.035157 | ─ | ─ | ─ | ─ | ─ | ─ | ─ | ─ | ─ | ─ | ─ | ─ |
| miR-505-3p | ─ | ─ | 0.66 | 0.02637 | 1.34 | 0.013671 | ─ | ─ | ─ | ─ | 0.79 | 0.00141 | ─ | ─ | ─ | ─ | ─ | ─ |
| miR-532-5p | ─ | ─ | ─ | ─ | 0.79 | 0.015777 | ─ | ─ | ─ | ─ | ─ | ─ | ─ | ─ | ─ | ─ | ─ | ─ |
| miR-543-3p | 0.96 | 0.02849 | ─ | ─ | 1.37 | 0.020281 | ─ | ─ | ─ | ─ | 0.90 | 0.00610 | ─ | ─ | ─ | ─ | ─ | ─ |
| miR-574-3p | 0.73 | 0.00568 | ─ | ─ | 1.22 | 0.023791 | ─ | ─ | ─ | ─ | 0.65 | 0.00900 | ─ | ─ | -0.90 | 0.04225 | ─ | ─ |
| miR-584-5p | 0.98 | 0.00462 | ─ | ─ | 1.32 | 0.022631 | ─ | ─ | ─ | ─ | 0.90 | 0.00407 | ─ | ─ | ─ | ─ | ─ | ─ |
| miR-589-5p | ─ | ─ | ─ | ─ | 0.82 | 0.014398 | ─ | ─ | ─ | ─ | ─ | ─ | ─ | ─ | ─ | ─ | ─ | ─ |
| miR-598-3p | ─ | ─ | ─ | ─ | 0.92 | 0.033575 | ─ | ─ | ─ | ─ | ─ | ─ | ─ | ─ | ─ | ─ | ─ | ─ |
| miR-625-3p | 0.96 | 0.02048 | ─ | ─ | 1.27 | 0.048522 | 0.65 | 0.019761 | ─ | ─ | 0.72 | 0.01095 | ─ | ─ | ─ | ─ | ─ | ─ |
| miR-654-3p | 1.16 | 0.0159 | ─ | ─ | 1.38 | 0.045322 | ─ | ─ | ─ | ─ | 0.91 | 0.01380 | ─ | ─ | ─ | ─ | ─ | ─ |
| miR-660-5p | ─ | ─ | ─ | ─ | 0.90 | 0.008458 | ─ | ─ | ─ | ─ | ─ | ─ | ─ | ─ | ─ | ─ | ─ | ─ |
| miR-664a-3p | ─ | ─ | 0.66 | 0.03730 | ─ | ─ | ─ | ─ | ─ | ─ | ─ | ─ | ─ | ─ | ─ | ─ | ─ | ─ |
| miR-7641-2-3p | -0.67 | 0.00546 | ─ | ─ | ─ | ─ | ─ | ─ | ─ | ─ | ─ | ─ | ─ | ─ | ─ | ─ | ─ | ─ |
| miR-769-5p | 0.68 | 0.03678 | ─ | ─ | 0.99 | 0.005218 | ─ | ─ | ─ | ─ | ─ | ─ | ─ | ─ | ─ | ─ | ─ | ─ |
| miR-885-5p | 0.98 | 0.02324 | ─ | ─ | 1.78 | 0.004457 | 0.83 | 0.002377 | ─ | ─ | 1.19 | 0.00006 | ─ | ─ | ─ | ─ | ─ | ─ |
| miR-92b-3p | ─ | ─ | ─ | ─ | 1.09 | 0.023582 | ─ | ─ | ─ | ─ | 0.76 | 0.00142 | ─ | ─ | ─ | ─ | ─ | ─ |
| miR-93-5p | ─ | ─ | 0.68 | 0.03968 | ─ | ─ | ─ | ─ | ─ | ─ | ─ | ─ | ─ | ─ | ─ | ─ | ─ | ─ |
| miR-98-5p | ─ | ─ | 1.04 | 0.03853 | ─ | ─ | ─ | ─ | ─ | ─ | ─ | ─ | ─ | ─ | ─ | ─ | ─ | ─ |
| miR-99a-5p | 0.62 | 0.03985 | 0.85 | 0.00805 | 1.52 | 0.003184 | ─ | ─ | ─ | ─ | 0.79 | 0.00368 | ─ | ─ | -0.99 | 0.00191 | ─ | ─ |
| miR-99b-5p | ─ | ─ | ─ | ─ | 1.16 | 0.010396 | ─ | ─ | ─ | ─ | 0.92 | 0.00012 | ─ | ─ | ─ | ─ | ─ | ─ |
