# Supplementary material for: Extracellular Vesicle Encapsulated MicroRNAs in Patients with Type 2 Diabetes Are Affected by Metformin Treatment
Source: J Clin Med. 2019 May 7;8(5):617. doi: 10.3390/jcm8050617 (PMC6571700; doi:10.3390/jcm8050617)
Supplement: Supplementary file 1 [file jcm-08-00617-s001.zip › jcm-499449-supppl/jcm-499449- Suppl figures.pdf]

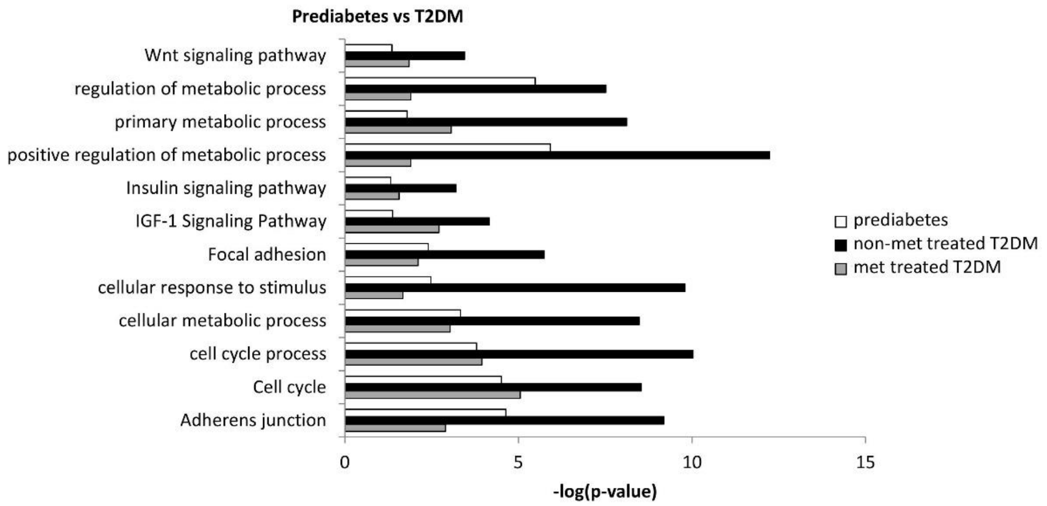

Figure S1: Fold enrichment of biological terms/pathways compared between METSIM and MetaHIT plasma miRNAs.

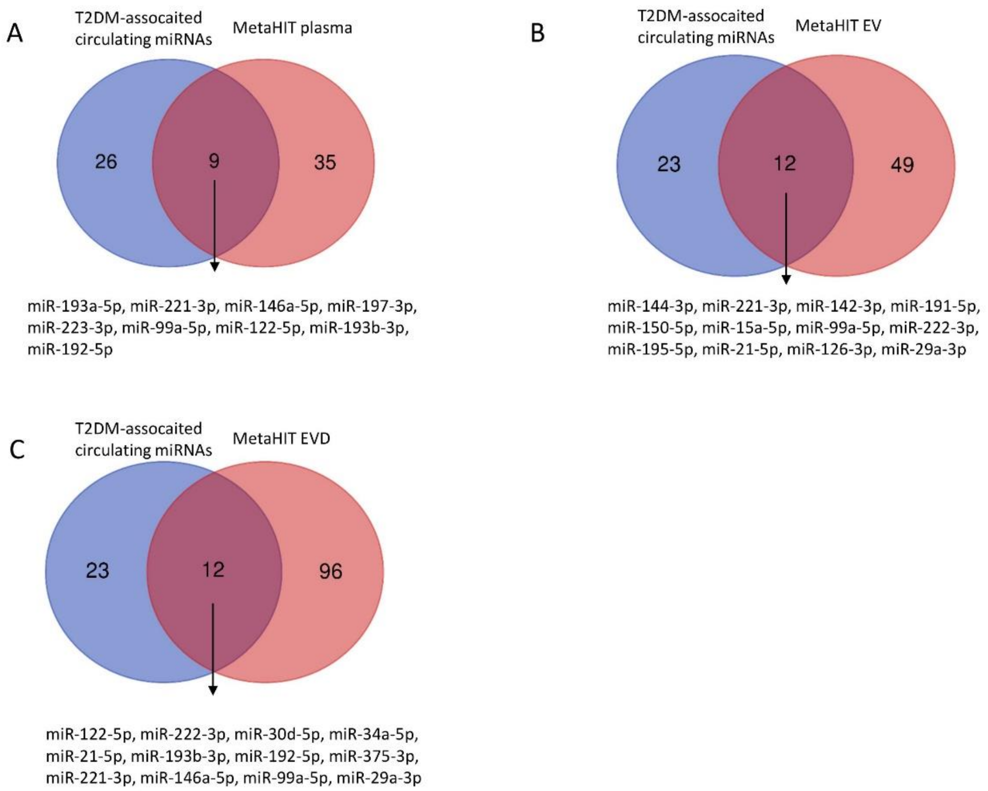

Figure S2: Overlap between circulating miRNAs associated with T2DM and circulating miRNAs identified from MetaHIT samples.
